# Supplementary material for: What contributes to good outcomes? The perspective of young people on short-term psychoanalytic psychotherapy for depressed adolescents
Source: PLoS One. 2021 Sep 24;16(9):e0257334. doi: 10.1371/journal.pone.0257334 (PMC8462705; doi:10.1371/journal.pone.0257334)
Supplement: Attachment — Submitted filename: Response to Reviewers PONE-D-21-07634.docx [file pone.0257334.s001.docx]

**Response to Reviewers**

Dear editorial team

Thank you for considering our paper and for the reviewers’ comments. The changes in the manuscript have been highlighted in yellow.

Please see our response to the comments below.

Best wishes,

Harriet Housby, Lisa Thackeray, Nick Midgley

_________

**Response to comments (in blue):**

| **In response to the academic editors’ comments:** | |
| --- | --- |
| 1. Please ensure that your manuscript meets PLOS ONE's style requirements, including those for file naming. The PLOS ONE style templates can be found at:      - https://journals.plos.org/plosone/s/file?id=wjVg/PLOSOne_formatting_sample_main_body.pdf - https://journals.plos.org/plosone/s/file?id=ba62/PLOSOne_formatting_sample_title_authors_affiliations.pdf | The manuscript has been edited to reflect these style template (to the best of our knowledge – see 'Revised Manuscript with Track Changes'). |
| 2. You indicated that you had ethical approval for your study. In your Methods section, please ensure you have also stated whether you obtained consent from parents or guardians of the minors included in the study or whether the research ethics committee or IRB specifically waived the need for their consent. | The methods section has been updated (p.12 L250-252) to state that we obtained consent from parents or guardians of the minors included in the study.  “The study was approved by the Cambridgeshire 2 Research Ethics Committee (reference 09/H0308/137) and local NHS provider trusts. All patients and their parents gave written informed consent.” |
| 3. We note that you have indicated that data from this study are available upon request. PLOS only allows data to be available upon request if there are legal or ethical restrictions on sharing data publicly. For more information on unacceptable data access restrictions, please see <http://journals.plos.org/plosone/s/data-availability#loc-unacceptable-data-access-restrictions>   a) If there are ethical or legal restrictions on sharing a de-identified data set, please explain them in detail (e.g., data contain potentially sensitive information, data are owned by a third-party organization, etc.) and who has imposed them (e.g., an ethics committee). Please also provide contact information for a data access committee, ethics committee, or other institutional body to which data requests may be sent. | In response to point 3. We have made relevant excerpts from the transcripts available in the main text of report, to provide evidence to support the data analysis. This is in line with the consent given by young people and their parents. Given the very confidential nature of the interview data it would not be possible to make it available on a public platform, but researchers may contact the chief investigator of the study, Nick Midgley, if they wish to access the data for research purposes. |
| **In response to the reviewer’s comments** | |
| 3.Have the authors made all data underlying the findings in their manuscript fully available?    The PLOS Data policy requires authors to make all data underlying the findings described in their manuscript fully available without restriction, with rare exception (please refer to the Data Availability Statement in the manuscript PDF file). The data should be provided as part of the manuscript or its supporting information, or deposited to a public repository. For example, in addition to summary statistics, the data points behind means, medians and variance measures should be available. If there are restrictions on publicly sharing data—e.g. participant privacy or use of data from a third party—those must be specified. | We have made relevant excerpts from the transcripts available in the main text of report, to provide evidence to support the data analysis. This is in line with the consent given by young people and their parents. Given the very confidential nature of the interview data it would not be possible to make it available on a public platform, but researchers may contact the chief investigator of the study, Nick Midgley, if they wish to access the data for research purposes. |
| **Reviewer #1 comments - Introduction:**  As the experience of the treatment is the focus of this study, I would find it helpful to elaborate more on the treatment (e.g. therapist stance, session structure) | The introduction has been edited to include greater focus on STPP session structure and therapeutic stance. (P4-5, L91-118) |
| **Reviewer #1 comments - Methods:**  Sampling:  - the authors hint at 4-6 participants being common for this type of analysis earlier in the text, which I think would be best placed in the sampling session including a little more detail for the rationale of choosing the number of 5 investigated here. | The rationale for selecting 5 adolescents to explore has moved to this section and expanded on (p9. L199-201) |
| Table 1 patient demographics:  - The information in the table refers to 29 sessions being offered in 3 cases, the text says “up to 28”, which was confusing to me. It would be helpful to be consistent with the numbers or add an explanation why they differ.  - It would be interesting to place the characteristics of the 5 patients into the broader characteristics of the adolescents receiving STPP – e.g. were they similar in their baseline MFQ scores and age? | This was an error on our part, due to an assessment session being counted - has been corrected to 28.  These details have been added (see L203-207). |
| **Reviewer #1 comments - Methods:**  Data analysis  - I would find it helpful to elaborate on the data analysis also in the text, at least naming the steps of the iterative process, otherwise it is my opinion that the text is not comprehensive enough without consultation of the table (e.g. “Themes in this study were selected”)  - L.234 illustrates ◊extracts illustrate | The data analysis has been elaborated on in the text by names the steps of the iterative process. (L231-234)  L.262 has been altered. |
| **Reviewer #1 comments - Results:**  I find the diverging results of Shelah and Mitch and the idea of the importance of adjusting to the psychoanalytic model very interesting and I can relate to the argument of it being a core element of the therapeutic process; however I cannot quite follow the argument as it is described in the text – could you elaborate here (with regard to the fact that they both still had good quantitative outcomes and at least Mitch continued to go)?  - Instead of “makes clear” (in lines 464, 504 and 506) I would recommend using a more cautious language when generalizing the individual adolescent’s experience (e.g. using “suggests”)  - In line 504, I was confused about the interpretation of trust being lost in Anayas therapy. For me, that was not implicated in the presented statements, could you elaborate?  - Line 507: I think the manuscript would benefit from an earlier introduction of a delineation of “successful therapeutic process” (which seems to be used for only three of the five therapies although they all had “good outcomes” – an issue that is not mentioned before the discussion section). | Selah’s quantitatively good outcome could reflect that she ‘cut-off’ from or ‘masked’ her depressive state to avoid continuing in therapy.    Alternatively, it could have been because she sought support externally in a friend and research assistant whom she could share her feelings with who she reported finding very helpful and continued to see after dropping out of therapy. (therapeutic effect of research).  Mitch appeared to continue attending therapy under the belief that she would ‘find the source’ of her symptoms. This hope motivated her to go although by her account this was never achieved.  The language has been altered to be more cautious in lines 495, 534 and 536.  Thank you for highlighting this, as reviewer #2 mentioned in this line Anaya should be “Selah”  See introduction Line 91-118 for delineation of “successful therapeutic process” |
| **Reviewer #1 comments - Discussion:**  - In addition to preventing ruptures, it seems to be the case in at least one of the therapies (Selah), that a rupture happened and was not repaired; In combination with her quite aversive statements (although her quantitative outcome was good), I would be interested in the discussion of her specific therapeutic process as successful or not, ideally additionally considering the therapist’s evaluation of her symptoms and therapeutic process (if this information Is available). Moreover, with regard to the growing literature on rupture and repair in therapy, I would add the need for repairing ruptures (instead of merely preventing them) as one important clinical implication of the study. | Whilst the authors do not have access to the therapist’s evaluation of her symptoms and therapeutic process, the authors have attempted to make their interpretation clearer; that her therapeutic experience is not conventionally ‘successful’. (i.e. there has not been a deep- rooted change her relationship to herself). And the wider implication that this has that quantitative outcome cannot be used solely as an indicator of when to end therapy.  The authors have included literature on the importance of repairing ruptures (L646-650) as an important clinical implication of the study (L570-572). |
| **Reviewer #2 comments –**  In terms of language, the article is well-organized and well-written, requiring only minor corrections:    Line 31: Sentence fragment  Line 85: Capitalize each of the first letter of cognitive behavior therapy for consistency  Line 170: “Equipping adolescent…are thought to contribute” should change into “Equipping adolescents…is thought to contribute”  Lines 465-468: awkward sentence structure  Line 504: Was “Anaya” meant to be “Selah”? | The corrections have been made. |
| Regarding the implications of this study might the authors consider including as an implication that the results could be used to amend/enrich the STPP protocol, especially with regards to the way the treatment is framed so that the adolescents can better understand what to expect? For instance, anxiety in STPP seems to be both potentially facilitating or hindering for service users. If service users were better informed about what to expect in STPP as part of the STPP protocol, could that mitigate the potentially hindering effect of anxiety and ease the adolescent's process of adjustment to the particular treatment model? | The authors welcome this suggestion.  See lines 674-680: The results could be used to amend/enrich the STPP protocol, especially with regards to the way the treatment is framed so that the adolescents can better understand what to expect |
